# Validating the Mild Behavioral Impairment Checklist in a Cognitive Clinic: Comparisons With the Neuropsychiatric Inventory Questionnaire

**DOI:** 10.1177/08919887221093353

**Published:** 2022-04-17

**Authors:** Sophie Hu, Scott Patten, Anna Charlton, Karyn Fischer, Gordon Fick, Eric E. Smith, Zahinoor Ismail

**Affiliations:** 1 Department of Community Health Sciences, 2129 University of Calgary, Calgary, AB, Canada; 2 Hotchkiss Brain Institute, 2129 University of Calgary, Calgary, AB, Canada; 3 Department of Clinical Neurosciences, 2129 University of Calgary, Calgary, AB, Canada; 4 Department of Psychiatry, 2129 University of Calgary, Calgary, AB, Canada; 5 O’Brien Institute for Public Health, 2129 University of Calgary, Calgary, AB, Canada

**Keywords:** Alzheimer’s disease, mild cognitive impairment, neuropsychology, cognitive decline, behavior

## Abstract

**Objective:**

To compare the utility of the Mild Behavioral Impairment-Checklist (MBI-C) and Neuropsychiatric Inventory Questionnaire (NPI-Q) to capture NPS in subjective cognitive decline (SCD), mild cognitive impairment (MCI), and dementia.

**Methods:**

In this cross-sectional memory clinic study, linear regression models compared MBI-C (n = 474) and NPI-Q (n = 1040) scores in relation to Montreal Cognitive Assessment (MoCA) score.

**Results:**

MBI prevalence was 37% in subjective cognitive decline, 54% in mild cognitive impairment, and 62% in dementia. Worse diagnostic status was associated with higher MBI-C and NPI-Q score (*P* < .001), lower MoCA (*P* < .001), and greater age (*P* < .001). Higher MBI-C (β −.09; 95% CI −.13, −.05) and NPI-Q (β −.17; 95% CI −.23, −.10) scores were associated with lower MoCA scores, with psychosis most strongly associated (β −1.11; 95% CI −1.56, −.65 vs β −1.14; 95% CI −1.55, −.73).

**Conclusions:**

The MBI-C captures global and domain-specific NPS across cognitive stages. Both the MBI-C and NPI-Q have utility in characterizing NPS.

The emergence of persistent neuropsychiatric symptoms (NPS) in later life is a marker of dementia risk. To examine the relationship between NPS and cognition, we compared the Mild Behavioral Impairment-Checklist (MBI-C) and Neuropsychiatric Inventory Questionnaire (NPI-Q) in participants with subjective cognitive decline (SCD), mild cognitive impairment (MCI), and dementia. In this cross-sectional study of 1238 participants recruited through an academic cognitive clinic, NPS severity was rated using the MBI-C (n = 474) and the NPI-Q (n = 1040). Analyses were conducted to compare scores on the MBI-C and the NPI-Q to scores on the Montreal Cognitive Assessment (MoCA), adjusting for differences in age, sex, education, and cognitive diagnosis. MBI was common in this specialty clinic sample, with 37% in SCD and 54% in MCI having MBI. In dementia, 62% had scores above MBI-C cutoff. More severe cognitive diagnostic status was associated with higher MBI-C and NPI-Q scores, lower MoCA scores, and greater age. Domain scores on both the MBI-C and NPI-Q were associated with lower MoCA scores. Among domains, psychosis was most strongly associated with lower MoCA score. The association between MBI-C and MoCA was modified by age, sex, and diagnosis. Directly comparing the MBI-C and the NPI-Q, there was a significant correlation between total global scores, although less so in SCD, compared to MCI and dementia. In this cognitive clinic sample, the MBI-C demonstrated the ability to detect NPS across cognitive stages and capture global as well as domain-specific NPS. Both the MBI-C and NPI-Q are useful for characterizing NPS, and complement one another to capture NPS across the cognitive spectrum.

## Introduction

Although memory loss is a hallmark symptom of dementia, neuropsychiatric symptoms (NPS) are recognized as core features that can emerge early in the disease course.^1^ Recent evidence suggests that 59% of people with dementia develop NPS before diagnosis of a cognitive disorder, including 30% of those who develop Alzheimer’s Disease (AD).^2^ Mild behavioral impairment (MBI) is a validated neurobehavioral syndrome characterized by later life emergence of persistent NPS, representing a change from longstanding patterns of behavior and personality, as an at-risk state for dementia.^3,4^ MBI is a non-cognitive maker of dementia,^5^ associated with incident cognitive decline and dementia,^6-11^ greater progression rate from mild cognitive impairment (MCI) to dementia, and lower reversion rate to normal cognition,^12^ AD risk genes,^13,14^ in vivo markers of amyloid, tau, and neurodegeneration,^15-20^ and 5-year post MBI neuropathological confirmation of AD.^21^ Assessing MBI may be a suitable approach to aid in early dementia detection.^22^

Neuropsychiatric symptoms have been historically described in dementia and measurement approaches have reflected that dementia focus, including well known scales such as the Neuropsychiatric Inventory Questionnaire (NPI-Q).^23^ The NPI-Q is a 12-item scale, which captures symptoms over a 1-month duration. The MBI Checklist (MBI-C),^24^ developed for non-dementia community-dwelling older adults, is the case ascertainment instrument developed to capture MBI in accordance with the MBI criteria. The MBI-C is a 34-question scale, which assesses NPS in the 5 MBI domains of apathy, mood/anxiety, impulse dyscontrol, social cognition, and psychosis. The MBI-C is explicit that symptoms emerge in later life and persist for at least 6 months.

As the MBI-C is a relatively new instrument, its ability to capture NPS requires further assessment. The objective of this study was to investigate the association between NPS and cognition in a cognitive neurology clinic sample of participants with subjective cognitive decline (SCD), MCI, and dementia, using the MBI-C and NPI-Q.

## Methods

### Data Collection

Participant data were obtained from the PROspective Registry of Persons with Memory SyMPToms (PROMPT), which enrolls participants referred for cognitive complaints from primary and specialty care to the Cognitive Neurosciences Clinic in Calgary, Alberta, Canada. PROMPT was established in 2010, with participants consenting use of the data captured in routine clinical care such as measures of cognition, behavior, and function. This registry has been utilized in research on caregiver burden, prevalence, and impulse dyscontrol in MBI^25,26^; comparisons of research and clinical cohorts with MCI^27^; and the impact of COVID on dementia care and well-being for patients and caregivers.^28^ This is a cross-sectional study design analyzing baseline scores. NPI-Q data were collected since 2010 and MBI-C since 2017. For a proportion of patients both were collected and administered in random order. For this study, data were used from the first clinic visit. Therefore, the data do not include any non-independent observations due to multiple ratings in the same subjects, and for each patient the diagnosis came from the same year as their other ratings.

### Standard Protocol Approvals, Registrations, and Participant Consents

Participants or their proxy provided written consent to be enrolled in PROMPT at their initial clinic visit. Ethics approval was obtained from the Conjoint Health Research Ethics Board at the University of Calgary (Ethics ID: REB17-1930).

### Neuropsychiatric and Cognitive Evaluations

The MBI-C and NPI-Q were completed by an informant (i.e., spouse/partner, family member, or caregiver) during one clinic visit, in no specific order. In both the MBI-C and NPI-Q symptoms are rated as present or absent, and if present are rated for severity as 1, 2, or 3, corresponding to mild, moderate, or severe symptoms. MBI-C and NPI-Q total severity scores were calculated by summing individual item severities, with absent symptoms assigned a zero score. For the MBI-C, domain scores were determined by summing severity scores for all questions in each domain. MBI cases were defined as total symptom severity >7, based on a preliminary validation study of primary care participants with cognitive complaints.^29^ Since the NPI-Q consists of 12 items, we applied a previously published transformational algorithm to map NPI-Q items onto MBI-C domains.^26,30^ NPI-Q neurovegetative items (night-time behaviors and appetite and eating) were excluded from the analysis as they do not map easily onto MBI criteria. As data were collected as part of clinical care, rating scales were administered in their original form without modification. Study aims were not to compare detection of MBI using both scales, but rather to assess in situ, the performance of both measures in capturing NPS. Thus, NPS were assessed over a 6-month reference range for the MBI-C and a 1-month reference range for the NPI-Q.

The Montreal Cognitive Assessment (MoCA)^31^ was administered and scored by a physician or clinic nurse. In accordance with standard MoCA scoring, an additional point was included in the final scoring for individuals with high school education or lower. Diagnosis (SCD, MCI, or dementia) was determined by a specialist physician based on appropriate criteria^32-34^ and work-up included neuropsychological testing (CERAD battery^35^), physical exam, and medical and social history.

### Statistical Analysis

Descriptive statistics for SCD, MCI, and dementia participants were reported using χ2 test (sex and education), one-way ANOVA when assumptions of normality, variance, and independence were met (age), and Kruskal–Wallis test for non-normal continuous variables (MBI-C, NPI-Q, and MoCA scores). Post-hoc analyses were performed for variables that significantly differed between cognitive groups. Tukey’s HSD was performed for age and education, as the assumption of homogeneity of variance was met through Levene’s test. Dunn’s test with Bonferroni correction was performed following Kruskal–Wallis testing. Descriptive statistics are presented without imputation (Tables 1 and 2).

**Table 1.** Demographics and Baseline MBI-C Scores by Cognitive Diagnosis for Complete Cases (n = 300).

Mean/Proportion (±SD/SE)	SCD (n = 52)	MCI (n = 79)	Dementia (n = 169)	Overall (n = 300)	Overall *P*-value	Post Hoc *P*-value		
						SCD vs MCI	MCI vs Dementia	SCD vs Dementia
Age	61.31 (9.76)	65.49 (9.64)	69.36 (9.37)	66.94 (9.96)	<.001	.038	.009	<.001
Sex (% female)	28 (53.8)	35 (44.3)	74 (43.8)	137 (45.7)	.427			
Education (% less than high school)	18 (34.6)	39 (49.4)	77 (45.6)	134 (44.7)	.236			
MBI-C Total Score	5.62 (7.27)	11.80 (12.73)	12.94 (12.17)	11.37 (11.90)	<.001	<.001	.373	<.001
MBI-C Apathy	1.44 (2.44)	3.14 (4.33)	3.53 (4.07)	3.07 (3.98)	.004	.032	.118	<.001
MBI-C Mood and Anxiety	2.02 (3.11)	3.39 (4.37)	3.59 (3.89)	3.26 (3.93)	.04	.028	.445	<.001
MBI-C Impulse Dyscontrol	1.75 (2.46)	3.97 (4.27)	4.08 (4.57)	3.65 (4.27)	.002	<.001	1.000	<.001
MBI-C Social Inappropriateness	.37 (.74)	.84 (1.81)	1.05 (2.06)	.88 (1.84)	.061	.503	.421	.066
MBI-C Psychosis	.04 (.19)	.46 (1.83)	.69 (1.36)	.52 (1.41)	.012	.135	.014	<.001
Montreal Cognitive Assessment Total Score	26.50 (2.48)	21.82 (4.25)	18.51 (5.71)	20.77 (5.74)	<.001	<.001	<.001	<.001

Abbreviations: SCD, subjective cognitive decline; MCI, mild cognitive impairment; MBI-C, mild behavioral impairment-checklist.

**Table 2.** Demographics and Baseline NPI-Q Scores by Cognitive Diagnoses for Complete Cases (n = 762).

Mean/Proportion (±SD/SE)	SCD (n = 147)	MCI (n = 210)	Dementia (n = 405)	Overall (n = 762)	Overall *P*-value	Post Hoc *P*-value		
						SCD vs MCI	MCI vs Dementia	SCD vs Dementia
Age	58.37 (10.43)	63.49 (9.65)	68.69 (10.01)	65.26 (10.77)	<.001	<.001	<.001	<.001
Sex (% female)	84 (57.1)	109 (51.9)	187 (46.2)	380 (49.9)	.059			
Education (% less than high school)	57 (38.8)	110 (52.4)	206 (50.9)	373 (49.0)	.022	.030	.032	.932
NPI-Q Total Score	2.84 (3.35)	4.54 (4.62)	5.52 (4.70)	4.73 (4.55)	<.001	<.001	.003	<.001
NPI-Q Apathy	.39 (.73)	.64 (.92)	.99 (1.04)	.78 (.98)	<.001	.018	<.001	<.001
NPI-Q Mood and Anxiety	1.17 (1.50)	1.77 (1.98)	1.81 (1.76)	1.67 (1.79)	.001	.008	.351	<.001
NPI-Q Impulse Dyscontrol	1.07 (1.48)	1.60 (1.93)	1.98 (2.03)	1.70 (1.94)	<.001	.019	.015	<.001
NPI-Q Social Inappropriateness	.18 (.51)	.39 (.81)	.45 (.82)	.38 (.77)	.002	.032	.309	<.001
NPI-Q Psychosis	.03 (.16)	.14 (.58)	.29 (.88)	.20 (.72)	<.001	.231	.010	<.001
Montreal Cognitive Assessment Total Score	25.86 (3.22)	22.19 (3.72)	18.59 (5.50)	20.98 (5.47)	<.001	<.001	<.001	<.001

Abbreviations: SCD, subjective cognitive decline; MCI, mild cognitive impairment; NPI-Q, neuropsychiatric inventory questionnaire

**Figure 1.**
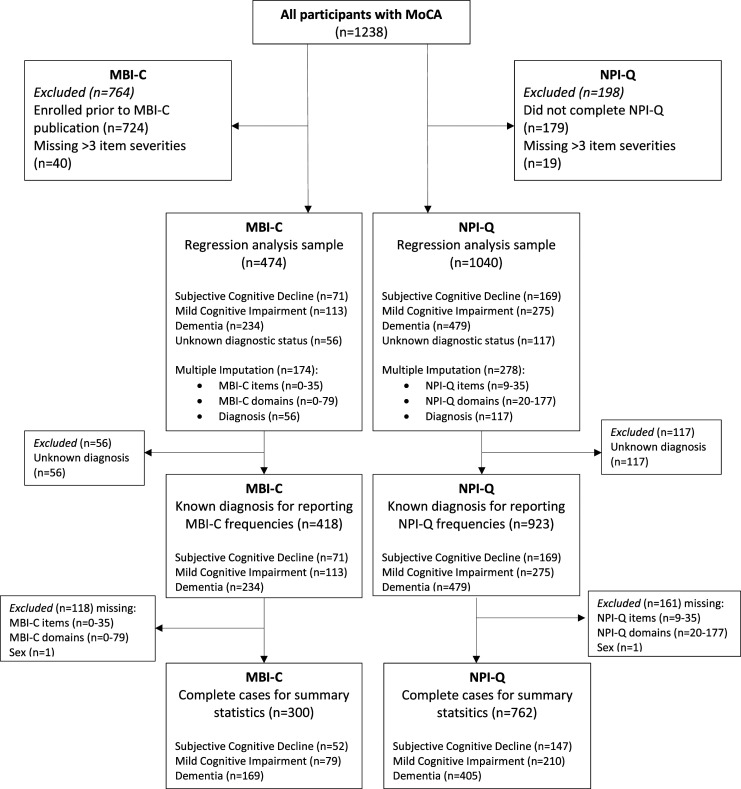
Flowchart of participants included for MBI-C and NPI-Q analysis. Complete cases were used for reporting baseline demographic and score statistics. Participants with a known diagnosis were used for reporting MBI-C and NPI-Q frequencies. Multiple imputation was used for the regression analyses samples.

For the MBI-C and NPI-Q, cases missing >3 item severities (out of a total of 34 possible item severities for the MBI-C and 10 for the NPI-Q) were excluded (Figure 1). Cases were stratified into those with diagnoses to determine MBI-C and NPI-Q frequencies and then further stratified into complete cases for summary statistics. Complete cases were the primary analyzed dataset for baseline demographic and score statistics. Remaining missing values for item severities underwent multiple imputation, which formed the dataset for regression analyses to compare associations between MBI-C and NPI-Q scores and cognition.

Observations were found to be missing at random when comparing variables of interest between completers and non-completers. To assess whether those with and without diagnoses differed, cross-tabulations with analyses of variance were completed, demonstrating no significant differences in both MBI-C and NPI-Q total and domain scores. For regression analyses, multivariate imputation chained equations with predictive mean matching (which combines linear regression and nearest neighbor imputation methods) was conducted. Imputation models included all variables used in analyses. Twenty imputed datasets were generated with 5 nearest neighbors. For the MBI-C, 37% (174/474) of participants had ≤3 missing questions which were imputed. For the NPI-Q, 27% (278/1040) of participants had ≤3 missing questions which were imputed. There were no missing observations for age or education. For MoCA score, only participants with complete MoCA data were included.

Forward stepwise linear regression models were developed to assess baseline MBI-C and NPI-Q total and domain scores in relation to MoCA total score. Age (years), sex (male/female), education (high school or more/less than high school), and diagnosis (SCD, MCI, dementia, or unknown) were assessed for modification and confounding. Separate models were run for each covariate independently with MBI-C or NPI-Q total and domain scores as exposure, and MoCA total score as outcome. Age was included in all models, including main effects models, as age significantly increased from SCD to MCI to dementia and scores were expected to vary with age. MoCA score was centered by median score given its left-skewed distribution. Modification was determined by β_3_ modification term (exposure*covariate) significance. Models with modification were stratified. Spearman’s correlations were implemented to assess the relationship between MBI-C and NPI-Q. All analyses were conducted in STATA 13.0.

### Data Availability Statement

Data used for the current study are available upon request to the corresponding author (ZI).

## Results

### Baseline Characteristics

Of the 474 participants with an MBI-C, 426 had MBI-C>0 and clinical diagnoses were made in 418/474 (88%). In the remainder, diagnoses were missing. Of those with diagnoses, 71 had SCD (17%), 113 had MCI (27%), and 234 had dementia (56%). Of complete cases (n=300), more impaired participants were older, with no significant difference in sex and education. Mean MoCA score decreased from 26.50 (SD ± 2.48) in SCD, to 21.82 (SD ± 4.25) in MCI, and 18.51 (SD ± 5.71) in dementia. Increasing severity of clinical diagnosis from SCD to MCI to dementia was associated with a higher MBI-C total score, with a mean of 5.62 (SD ± 7.27) in SCD, 11.80 (SD ± 12.73) in MCI, and 12.94 (SD ± 12.17) in dementia. Increasing severity of clinical diagnosis was also associated with higher domain scores for apathy, mood/anxiety, impulse dyscontrol, social inappropriateness, psychosis, and lower MoCA score. In *post hoc* analyses, the most significant differences were seen between SCD and MCI participants, and between MCI and dementia participants (see Table 1 for statistical details).

Of the 1040 participants with an NPI-Q, clinical diagnoses were made in 923/1040 (89%). Of those with diagnoses, 169 had SCD (18%), 275 had MCI (30%), and 479 had dementia (52%). Of complete cases (n=762), more impaired participants were older and had less than high school education, with no significant difference in sex. Mean MoCA score decreased from 25.86 (SD ± 3.22) in SCD, to 22.19 (SD ± 3.72) in MCI, and 18.59 (SD ± 5.50) in dementia. Increasing severity of clinical diagnosis from SCD to MCI to dementia was associated with a higher mean NPI-Q total score, with a mean of 2.84 (SD ± 3.35) in SCD, 4.54 (SD ± 4.62) in MCI, and 5.52 (SD ± 4.70) in dementia. Increasing severity of disease status was also associated with higher domain scores for apathy, mood/anxiety, impulse dyscontrol, social inappropriateness, and psychosis, and lower MoCA score. In *post hoc* analyses, the most significant differences were seen between SCD and MCI participants, and between MCI and dementia participants (see Table 2 for statistical details).

### Mild Behavioral Impairment-Checklist and Neuropsychiatric Inventory Questionnaire Total and Domain Frequencies

**Figure 2.**
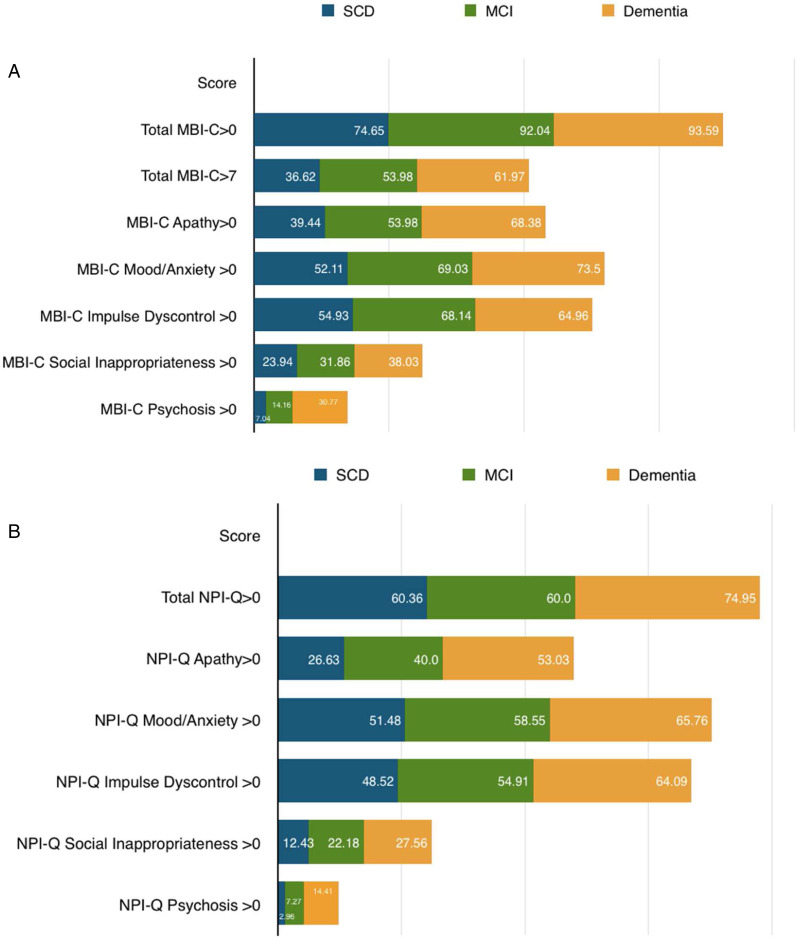
Non-imputed counts of MBI-C (a) and NPI-Q (b) severity scores by diagnosis. MBI-C overall scores include >0 and >7 (cutpoint).

Overall, 90% of participants had an MBI-C total score>0 (426/474) while 55.91% (265/474) scored above the MBI cutpoint of 7. Of those with diagnoses, 90% had MBI-C>0 (376/418) and 55.5% (232/418) had MBI-C>7. Of those with SCD, 36.62% (26/71) had MBI, and 53.98% (61/113) of MCI participants had MBI (Figure 2A). In those with dementia, 61.97% (145/234) scored above the MBI cutpoint. Over half of participants had apathy (249/418; 59.57%), mood/anxiety (287/418; 68.66%), or impulse dyscontrol (268/418; 64.11%), while fewer had social inappropriateness (142/418; 33.97%) or psychotic symptoms (93/418; 22.25%). Overall, 67.98% of participants had an NPI-Q total score>0 (707/1040). Of those with diagnoses, 60.36% of SCD (102/169), 60.00% of MCI (165/275), and 74.95% of dementia (359/479) had NPI-Q>0 (Figure 2B). Over half of participants had mood/anxiety (563/923; 61%) or impulse dyscontrol (540/923; 58.50%), while fewer had apathy (409/923; 44.31%), social inappropriateness (214/923; 23.19%), or psychosis (94/923; 10.18%). Similar trends were observed for each participant group.

### Association Between Mild Behavioral Impairment-Checklist Score and Cognition

Each of the domains, when considered alone, were significantly associated with lower MoCA score (Table 3). For associations with modifiers, stratified results are presented. Psychosis was most strongly associated with MoCA. For those over 66 years old, a one-point higher psychosis score was associated with a 1.11-point lower MoCA score (95% CI −1.56, −.65, R^2^ = .12). For mood/anxiety, being male was associated with a .31-point lower MoCA score (95% CI −.46, −.15, R^2^ = .11), while for females, mood/anxiety was not significantly associated with MoCA (β −.077, 95% CI −.27, .11 R^2^ = .16). SCD (β −.37, 95% CI −.58, −.16, R^2^ = .24) and MCI (β −.21, 95% CI −.40, −.02, R^2^ = .07) participants with impulse dyscontrol had a greater association with low MoCA score than dementia participants (β .011, 95% CI −.13, .15, R^2^ = .03).

**Table 3.** Linear Regression Models With MOCA Score as Outcome and Assessment of Modification^a^ for the MBI-C (n = 474) and NPI-Q (n = 1040).

Models	MBI-C Final Model				NPI-Q Final Model			
	β_1_	SE	95% CI	*P*-value	β_1_	SE	95% CI	*P*-value
Total	−.09	.02	−.13, −.05	<.0001	−.17	.03	−.23, −.10	<.0001
Apathy	−.21	.06	−.32, −.09	<.0001	−.42	.16	−.73, −.10	.010
Mood and anxiety	–.31 (Male)	.078	−.46, −.15	<.0001	−.30	.09	−.48, −.13	.001
	–.077 (Female)	.096	−.27, .11	.425				
Impulse dyscontrol	–.37 (Subjective cognitive decline)	.10	−.58, −.16	.001	−.33	.08	−.48, −.17	.0001
	–.21 (MCI)	.096	−.40, −.02	.034				
	.011 (Dementia)	.070	−.13, .15	.875				
Social Inappropriateness	–.33	.13	−.59, −.08	.010	−.34	.20	−.74, .06	.100
Psychosis	–1.11 (age >=66)	.23	−1.56, −.65	<.0001	−1.14	.21	−1.55, −.73	<.0001
	–.43 (age <66)	.27	−.96, .10	.109				

MBI-C models for assessment of modification are as follows.

Crude: E(MoCA)= β_0_ + β_1_MBI + β_2_Age.

Age: E(MoCA)= β_0_ + β_1_MBI + β_2_Age + β_3_Age*MBI.

Sex: E(MoCA)= β_0_ + β_1_MBI + β_2_Age + β_3_Sex + β_4_Sex*MBI.

Education: E(MoCA)= β_0_ + β_1_MBI + β_2_Age+ β_3_Education + β_4_Education*MBI.

Diagnosis: E(MoCA)= β_0_ + β_1_MBI + β_2_Age+ β_3_Diagnosis + β_4_Diagnosis*MBI.

NPI-Q models for assessment of modification are as follows.

Crude: E(MoCA)= β_0_ + β_1_NPI+ β_2_Age.

Age: E(MoCA)= β_0_ + β_1_NPI + β_2_Age + β_3_Age*NPI.

Sex: E(MoCA)= β_0_ + β_1_NPI + β_2_Age + β_3_Sex + β_4_Sex*NPI.

Education: E(MoCA)= β_0_ + β_1_NPI + β_2_Age + β_3_Education + β_4_Education*NPI.

Diagnosis: E(MoCA)= β_0_ + β_1_NPI + β_2_Age + β_3_Diagnosis + β_4_Diagnosis*NPI.

Abbreviations: MOCA, Montreal Cognitive Assessment; MCI-C; Mild Behavioral Impairment-Checklist; MCI, mild cognitive impairment; NPI, neuropsychiatric inventory; NPI-Q, neuropsychiatric inventory questionnaire

^a^Final models are presented by strata if modification was found.

### Association Between Neuropsychiatric Inventory Questionnaire and Cognition

All domains except social inappropriateness were significantly associated with a lower MoCA score (Table 3). Psychosis was most strongly associated with MoCA. A one-point higher psychosis score was associated with a 1.14-point lower MoCA score (95% CI −1.55, −.73, R^2^ = .15). Apathy (β −.42, 95% CI −.73, −.10, R^2^ = .13), mood/anxiety (β −.30, 95% CI −.48, −.13, R^2^ = .13), impulse dyscontrol (β −.33, 95% CI −.48, −.17, R^2^ = .13), and social inappropriateness (β −.34, 95% CI −.74, .06, R^2^ = .12) were associated with a less than one-point lower MoCA score. Given that no modification was found, main effects models were presented.

### Correlation Between Mild Behavioral Impairment-Checklist and Neuropsychiatric Inventory Questionnaire

The MBI-C and NPI-Q total and domain scores were significantly positively correlated as determined by Spearman’s correlation testing. In the whole sample, the correlation between MBI-C and NPI-Q total score was .67 (ρ(227), *P* < .00001), and cognitive category correlations were .58 (ρ(38), *P* = .0001) in SCD, .68 (ρ(47), *P* < .00001) in MCI, and .65 (ρ(138), *P* < .00001) in dementia. For apathy, whole sample correlation was .56 (*P* < .00001), and for cognitive categories correlations were .62 (ρ(38), *P* < .00001) in SCD, .65 (ρ(47), *P* < .00001) in MCI, and .49 (ρ(138), *P* < .00001) in dementia. For mood/anxiety whole sample correlation was .57 (*P* < .00001), and for cognitive categories correlations were .55 (ρ(38), *P* = .0002) in SCD, .55 (ρ(47), *P* < .00001) in MCI, and .57 (ρ(138), *P* < .00001) in dementia. For impulse dyscontrol whole sample correlation was .61 (*P* < .00001), and for cognitive categories correlations were .49 (ρ(38), *P* = .0014) in SCD, .65 (ρ(47), *P* < .00001) in MCI, and .60 (ρ(138), *P* < .00001) in dementia. For social inappropriateness whole sample correlation was .51 (*P* < .00001), and for cognitive categories correlations were .40 (ρ(38), *P* = .0104) in SCD, .37 (ρ(47), *P* = .0085) in MCI, and .55 (ρ(138), *P* < .00001) in dementia. For psychosis whole sample correlation was .48 (*P* < .00001), and for cognitive categories correlations were .70 (ρ(38), *P* < .00001) in SCD, .23 (ρ(47), *P* = .1063) in MCI, and .49 (ρ(138), *P* < .00001) in dementia.

## Discussion

Consistent with previous literature, we found that NPS were common, with a high NPS frequency but low symptom severity, worse with a more impaired diagnostic status.^36-39^ Both the MBI-C and the NPI-Q determined that NPS were frequently observed in all cognitive categories, in this memory clinic sample. Measured with the MBI-C, the majority of participants had at least 1 NPS (89.87% overall, 74.65% SCD, 92.04% MCI, and 93.59% dementia). Similarly, measured with the NPI-Q, the majority of participants had at least 1 NPS (67.98% overall, 60.36% SCD, 60.00% MCI, and 74.95% dementia).

Using a previously determined general MBI-C cutpoint of >7, prevalence of MBI overall was 55.50%. MBI prevalence increased with more impaired diagnostic status from 36.62% in SCD to 53.98% in MCI. In dementia, 61.97% of participants scored above cutpoint. A systematic review and meta-analysis of 11 studies including 15,689 participants estimated pooled prevalence of MBI to be 33.5%. Stratified by cognitive category, prevalence of MBI was estimated at 45.5% in MCI, 35.8% in SCD, and 17.0% in cognitively normal older adults.^40^ For the MBI-C, we are not aware of comparable studies in a quaternary-level memory clinic sample, but a recently published study of prevalence of MBI in an Iranian memory clinic found 50% of patients with MCI had MBI at a cutpoint of ≥7 (Kianimehr 2021).^41^ Our own work has demonstrated that NPS are more prevalent in clinical versus community samples.^42^ Thus, in our sample the MBI prevalences of 37% in SCD and 54% in MCI are felt to be accurate representations in this sample, providing some reassurance in the methodology. Importantly, a general cutpoint was used, and domain-specific cutpoints have not yet been explored. Psychosis, for example, would confer great risk of dementia, even if it were the only domain endorsed with a total MBI-C score below threshold. More research is required to explore this important issue.^43^

With the NPI-Q, there is no established cutpoint, and MBI domains were derived using transformed NPI-Q scores. In a prior PROMPT study, higher MBI prevalence was observed in MCI (85.3%) compared to SCD (76.5%), with a liberal cutpoint of >0.^26^ In the PATH study, a population cohort drawn from an electoral roll, NPI-based prevalence estimates for MBI were 34.1% globally, 47.1% in SCD, and 53.4% in MCI.^30^ A 1-month reference range was used in both of these studies. With shorter reference ranges, transient or reactive NPS may reflect short-term adjustment to life events rather than the chronic effects of neurodegeneration. These shorter reference ranges may result in inflated prevalence estimates, less specific for behavioral manifestations of neurodegenerative diseases such as AD.

Indeed, variable MBI diagnostic approaches are a major contributor to heterogeneity in estimates of prevalence of global MBI and domains.^40,44^ As the MBI-C is a novel instrument, not yet available in cohorts like the Alzheimer’s Disease Neuroimaging Initiative or National Alzheimer’s Coordinating Center, variable symptom durations and NPS measures have been used in many studies. More sensitive and specific prevalence estimates are expected as more MBI-C data become available. In the previous PROMPT study, prevalence estimates for mood/anxiety (77.8%), impulse dyscontrol (64.4%), and apathy (51.7%) were highest, while social inappropriateness (27.8%) and psychosis (8.7%) were lowest.^26^ Recent studies of MBI prevalence with the MBI-C, from a Spanish rural primary care clinic sample using conservative criteria for MBI classification, found 5.8% of SCD participants^45^ and 14.2% of MCI participants^46^ had MBI. In a population based cohort of 9931 participants in the UK PROTECT study, MBI prevalence was determined to be 10% in this cognitively normal community sample.^6^ In a similar cohort from the Brain Health Registry of 499 largely cognitively unimpaired participants who were able to complete online neuropsychological testing, MBI prevalence was 6.2% at an MBI-C cutoff >7.^47^ These lower prevalences likely better reflect the baseline rate in primary care and community samples, given the MBI-C was used for case ascertainment. Additionally, community samples likely have fewer cognitively impaired individuals than the specialty care cognitive neurology population represented here. These findings reflect the possibility that the presence of NPS and cognitive complaints prompt presentation to specialty care.^42^

With respect to MBI-C scores in dementia, although MBI is a pre-dementia construct, researchers have used the MBI-C in dementia populations.^48,49^ In a validation study of the Chinese translation of the MBI-C, the MBI-C demonstrated good internal consistency reliability, test–retest reliability, and inter-rater reliability. Its optimal cutoff point was 6/7 for identifying AD dementia, with a sensitivity of 86.96% and specificity of 86.00%, and its detection rate for moderate–severe AD dementia was higher than that of the NPI-Q. The authors indicated the Chinese version of the MBI-C has high validity as a screening tool for AD dementia and confirmed the significance of the MBI-C in differentiating the severity of dementia.^48^

The MBI-C and NPI-Q were comparable with respect to greater NPS burden associated with impaired cognition. All MBI-C domains were significantly associated with lower MoCA, and all but social inappropriateness measured with the NPI-Q were associated with lower MoCA as well. Psychosis had the strongest association with lower MoCA for both instruments (β −1.11, 95% CI −1.56, −.65 vs β −1.14, 95% CI −1.55, −.73). For both the MBI-C (β −.09; 95% CI −.13, −.05, R^2^ = .13) and NPI-Q (β −.17; 95% CI −.23, −.10, R^2^ = .14), total score was a weaker indicator of lower MoCA compared to individual domains. These findings support the utility of MBI domains, in addition to global MBI burden, for association with cognitive scores or status, incorporating age, sex, and diagnosis as modifiers for the relationship. 

In systematic reviews of NPS prevalence in MCI using the NPI-Q, prevalence has ranged from 35–85%.^50,51^ Depression, apathy, anxiety, and irritability were most frequent.^50,51^ We found that apathy and mood/anxiety, in addition to impulse dyscontrol, were prevalent in over half of participants. These relative frequencies are consistent with a prior Korean study of the MBI-C administered to a clinic sample comparing normal cognition, SCD, and MCI participants. MCI participants had higher scores for apathy, impulse dyscontrol, and total score, while mood/anxiety, psychosis, and social inappropriateness did not differ.^52^

Apathy has been found to be the most common NPS in dementia.^53^ Apathy occurred in 59.92% of individuals using the MBI-C, compared to less than half of individuals as measured by the NPI-Q (43.94%). The MBI-C was developed to capture the apathy subdomains of cognitive (decreased interest), behavioral (decreased initiative), and emotional (decreased emotional reactivity) apathy. This broader syndromic approach to apathy may have conferred greater sensitivity resulting in higher apathy prevalence when measured by the MBI-C. Incorporating the 3 apathy subdomains in the MBI-C is consistent with the structure of the recently revised criteria for syndromic apathy in neurodegenerative disease.^54^ In a recent MBI-C exploratory factor analysis,^55^ all apathy questions loaded on to the apathy domain, suggesting potential utility of the MBI-C for apathy.

Mood/anxiety captures low mood, anhedonia, hopelessness, guilt, worry, and panic. Mood/anxiety was the most prevalent domain as determined by both the MBI-C (68.57%) and NPI-Q (61.63%). The mood/anxiety domain was developed based on core symptomology of major depression and generalized anxiety. However, psychiatric constructs developed for the general population might not accurately reflect NPS as they emerge in older adults, perhaps assisting with interpretation of mixed findings for dementia markers in relation to conventional measures of psychiatric disorders.^56^ There is a paucity of research on sex and gender differences in neurodegeneration^57^ and these differences, in conjunction with NPS, should be further explored as risk factors for cognitive decline and dementia. Sex differences have been previously reported in cognitively normal, SCD, and MCI participants with males experiencing impulse dyscontrol and apathy more frequently than females.^30^ For affective symptoms, epidemiological studies of major depressive disorder established that females have higher rates of depression.^58^ Similarly, sex differences are observed in the associations with the mood/anxiety domain and MoCA score. Males with mood/anxiety had a significantly lower MoCA (β −.31; 95% CI −.46, −.15). Although females had a non-significant lower MoCA (β −.077; 95% CI −.27, .11), the direction of effect was the same as for males.

Impulse dyscontrol is defined as a loss of ability to delay gratification and control behavior, impulses, or oral intake and contains items related to agitation, aggression, impulsivity, recklessness, abnormal reward, and reinforcement.^3,20,59^ Over half the participants had impulse dyscontrol as 63.92% of MBI-C and 58.27% of NPI-Q participants displayed at least one symptom. Diagnostic status was a modifier with SCD and MCI status showing a significant association with lower MoCA. This suggests impulse dyscontrol may be a robust domain specifically for non-dementia participants.^25^ Differences in agitation, irritability, and aberrant motor behavior prevalence have been observed between participants with differing severities of AD.^52^ Although agitation and impulsivity have predominantly been studied in dementia, emerging studies demonstrate its prevalence and utility as a predictor of dementia in MCI and normal cognition,^10,59,60^ associated with AD diffusion tensor imaging markers.^20^ The MBI-C impulse dyscontrol domain has recently been explored in a network analysis, which assesses the network structure of a group of variables by measuring the conditional relationships between variables using partial correlations. Stubbornness/rigidity, agitation/aggressiveness, and argumentativeness were frequent and the most central symptoms in the network. Impulsivity, the fourth most central symptom in the network, served as the bridge between these common symptoms and less central and rare symptoms.^25^ Future studies focusing on impulse dyscontrol as a predictor of incident cognitive decline and dementia would further elucidate the role of this domain, and its relationship to the presence and measurement of syndromic agitation in dementia.^61,62^

Social inappropriateness describes not following societal norms and lacking social graces, tact, and empathy.^24^ Social inappropriateness as measured by the MBI-C was significantly associated with lower MoCA score (β −.33; 95% CI −.59, −.08). However, social inappropriateness as measured by the NPI-Q was not significantly associated with MoCA score (β −.34; 95% CI −.74, .06). The social inappropriateness domain of the NPI-Q consists of 1 item that encompasses both impulsivity (“Does the participant seem to act impulsively?”) and lack of social graces (“Does the participant talk to strangers as if he or she knows them, or does the participant say things that may hurt people’s feelings?”). Thus, there is no encompassing measure of social inappropriateness in the NPI-Q, as it is conflated with impulsivity, and in our study it was not a significant indicator of impaired cognition as measured by the MoCA. In comparison, the MBI-C captures social inappropriateness with 5 items on sensitivity, empathy, and tact, perhaps accounting for the significant association with MoCA score.

Psychosis is characterized by delusions and hallucinations and is known to be strongly associated with, and predictive of, onset of cognitive decline.^43,63,64^ We found that psychosis was the strongest and most significantly associated domain with cognition for both the MBI-C and NPI-Q. Psychosis was the least frequent domain and had low severity ratings; thus irrespective of severity, psychotic symptoms were associated with impaired cognition in this cognitive clinic population. Our findings are consistent with the literature. One study observed that the most distinguishing feature between pre-dementia and dementia participants was delusions.^37^ A cross-sectional study of the NPI-Q found that participants with delusions were 8 times more likely to have MCI than normal cognition, with delusions also having low prevalence of 9%.^36^ More recently, in a French prospective study, of all NPS, delusions were associated with the highest odds ratio for incident dementia at 10 years.^65^ The importance of psychosis in pre-dementia populations is increasingly being established, and new International Society to Advance Alzheimer’s Research and Treatment (ISTAART) research diagnostic criteria for psychosis in AD and related disorders include the emergence of psychosis in dementia-free older adults as a potential indicator of preclinical or prodromal AD.^66^ Similarly, the revision of the International Psychogeriatric Association (IPA) criteria for psychosis in neurocognitive disorders now extends beyond clinical dementia to include MCI.^67^ Psychosis is known to be age-dependent and emerges later in disease course.^63^ One study found that hallucinations were more common in MCI participants over 65.^68^ We found that psychosis as measured by the MBI-C had a negative association with cognition in older individuals (those over 66). Although we did not observe significantly impaired cognition in those under 66, the direction of effect was the same.

The correlation findings provide further insight into the similarities and differences between the MBI-C and NPI-Q. While correlations were moderate to high for total scores between scales, these correlations were lower in SCD, compared to MCI and dementia, possibly reflecting the a priori goal of the MBI-C to capture NPS in pre-dementia populations. Domain correlations varied, but generally domains correlated less well in SCD, versus MCI or dementia, similar to the total score correlations. The relatively low correlation of the scales for apathy in dementia is unexpected. This difference may reflect the fact that the MBI-C has 6 apathy questions, which were included with the explicit purpose of assessing the 3 apathy domains of decreased interest, initiative, and emotional reactivity.^54^ A validation of the MBI-C in an AD sample revealed a Cronbach’s α of .88 for this domain,^48^ suggesting good internal consistency of these apathy questions. These findings suggest that MBI-C apathy should be explored further in dementia. For mood/anxiety, the similar but moderate correlations between the MBI-C and NPI-Q suggest that affective symptoms are captured comparably across the cognitive continuum. For impulse dyscontrol the modest correlation in SCD is of interest, given that impulse dyscontrol does emerge early in the course of neurodegenerative disease.^59^ The MBI-C has a robust assessment of agitation, aggression, impulsivity, and abnormal reward salience, providing utility in capturing early behavioral manifestations of neurodegenerative disease.^25^ This domain may be considered a leading candidate for early detection and clinical trial sample enrichment^69^ due to the relatively high frequency of these symptoms and the association with AD proteinopathies. In the Swedish BioFinder2 study, for example, MBI-C measured impulse dyscontrol was associated with both CSF tau and tau-PET (in Braak Stage I–II regions) in cognitively normal Aβ+ individuals (i.e., preclinical AD). Symptoms in this domain can also be easily overlooked and normalized in older adults, warranting further education regarding new onset and persistent argumentativeness, irritability, impulsivity, frustration intolerance, reckless driving, stubbornness, rigidity, and repetitive or compulsive behaviors.^24^ The social inappropriateness domain occurs in low frequency in AD and related dementias, but was included in MBI criteria specifically to capture early frontotemporal dementia (FTD). Few studies have explored this domain, but in a study of motor neuron disease, related to FTD, a diagnosis of motor neuron disease with cognitive impairment was significantly more common in MBI, and social inappropriateness was the domain with the third greatest magnitude of cognitive decline over 15 months.^70^ The dearth of data for the MBI-C in FTD is a needs area for further study. For psychosis, interpreting the correlations is challenging, notwithstanding the fact that psychosis is common in AD but substantially less common in SCD. Given the magnitude of association between psychotic symptoms and cognitive impairment in our study, and the substantial risk for incident cognitive decline and dementia in dementia-free samples with new onset psychosis,^43,63^ this domain warrants further study.

Given that study recruitment was from a quaternary care clinic composed of participants referred for cognitive complaints, the results should only be cautiously applied to primary care samples or community population-based samples. Although biomarkers were not used for diagnostic confirmation, the vast majority of patients had AD and related dementias. While non-AD dementias are likely overrepresented in this memory clinic sample compared to clinical samples from long-term care, primary care, or the community, it is unknown whether our findings are applicable to FTD or Lewy body dementia. However, whereas the severity of cognitive problems may differ across these populations there is no reason to believe that the association between NPS burden and diagnostic group should differ. Therefore, we believe the results remain valid and applicable to other clinical populations. The sample size for the NPI-Q (n = 1040) was larger than the MBI-C (n = 474) because the MBI-C was only developed in 2017. This sample size difference confers greater statistical power and precision for the NPI-Q analyses than the MBI-C analyses, which should be kept in mind when interpreting the results. Cutpoints for MBI domains and MBI status using the NPI-Q remain to be determined. We examined independent associations between MBI-C domains and overall cognition. Interaction between domains will be explored in future analyses. This study is primarily descriptive. There were many *P*-values arising from the analysis and some may represent type I error. These findings should be considered exploratory unless (or until) they are replicated by other studies. Additional limitations are inherent with the use of a stepwise regression, including possible exclusion of important variables due to weak correlations with the dependent variable, and issues with multiple hypothesis testing. As an exploratory analysis, this approach provided foundational work, to be expanded upon in future studies.

## Conclusion

To our knowledge, this is the first study in a memory clinic sample to determine the association between NPS and cognition using the MBI-C and NPI-Q. Overall, the MBI-C and NPI-Q were comparable in capturing NPS broadly, but correlations between scales were lower in SCD compared to MCI and dementia, with some domain differences as well. Higher scores on both scales were associated with poorer cognition. The MBI-C and NPI-Q differed when capturing prevalence of MBI, with the MBI-C generating more conservative estimates. Apathy, depression, anxiety, and irritability are known risk factors for cognitive impairment.^51^ We found that less frequently occurring NPS including psychosis and social inappropriateness were stronger indicators of impaired cognition when participants presented with these symptoms, emphasizing the importance of MBI domains. The MBI-C is age-, sex-, and diagnosis-specific in its association with cognition; thus, these variables are important to include in risk models that include MBI. The mechanisms underlying these differences are unclear and should continue to be explored as they may have differing underlying pathologies requiring unique treatment. Overall, we observed significant differences in cognition and NPS between early phase pre-dementia participants with SCD/MCI and participants with dementia, suggesting that NPS should be considered in models of early phase dementia. The MBI-C is a valid measure of NPS and can be used in conjunction with the NPI-Q to capture NPS across the cognitive spectrum.
